# Cancer Diagnostic and Predictive Biomarkers 2016

**DOI:** 10.1155/2017/7362721

**Published:** 2017-06-18

**Authors:** Franco M. Buonaguro, C. David Pauza, Maria Lina Tornesello, Pierre Hainaut, Renato Franco, Massimo Tommasino

**Affiliations:** ^1^Unit of Molecular Biology and Viral Oncology, Department of Research, Istituto Nazionale Tumori, IRCCS, Fond. Pascale, 80131 Napoli, Italy; ^2^Institute of Human Virology, University of Maryland Medical School, Baltimore, MD 21201, USA; ^3^Department of Research, Institute for Advanced Biosciences, Grenoble Alpes University (UGA), 38700 La Tronche, France; ^4^Department of Pathology, Università della Campania “Luigi Vanvitelli”, 80138 Napoli, Italy; ^5^Infections and Cancer Biology Group, IARC, 150 Cours Albert Thomas, 69372 Lyon Cedex 08, France

Early diagnosis and prediction of therapeutic responses are crucial in cancer patients to tailor and optimize the treatment, increase the likelihood of cure, reduce side-effects, and avoid overtreatment. This special issue compiles relevant articles focused on the development of innovative cancer biomarkers and their validation.

New molecular biology tools, including genome-wide analysis, deep-sequencing, and RNA-seq, are currently available for the identification of novel unique biomarkers with great potential for developing more sensitive and specific diagnostic tools as well as discovering new candidate therapeutic targets for personalized medicine.

In this special issue the relevance of systemic biomarkers for early diagnosis and/or cancer characterization/monitoring has been emphasized by several articles focused on blood tumor markers with an overview by S. Holdenrieder et al. The great variety of blood tumor markers is shown by the wide range of biomarkers spanning from the basic parameter changes of blood count in renal cell carcinoma (reported by G. Prokopowicz et al.) to the detection of circulating tumor cells in gastric cancer (observed by K. Zou et al.), the level of antibodies to the hidden IgG antibodies to the tumor-associated Thomsen-Friedenreich antigen (described in gastric cancer patients by O. Kurtenkov and K. Klaamas), and the levels of plasma circulating tumor DNA, observed in melanoma patients by B. Busser et al. Moreover, the diagnostic relevance of systemic miRNAs (in particular miRNA 21) in biliary tract cancer seems even greater than current markers (i.e., CA19-9) and has been reported by C. Mayr et al.

Most articles, however, have been focused on tissue biomarkers in the early discovery and/or characterization stage, with a great potentiality of becoming relevant cancer biomarkers for molecular characterization/diagnosis, prognostic evaluation, and therapeutic responsivity. The articles on the predictive positive clinical significance of novel long noncoding RNA in HCC (by L. Zhang et al.) whose expression levels are directly related to overall survival, the low proliferation level of the human lung cancer cell lines A549 in presence of high expression of circular RNA-ITCH (by L. Wan et al.), and limited disease in SCLC of patients with miRNA-related polymorphisms in P13K/Akt/mTOR pathway genes (by W. Jiang et al.) are establishing a new paradigm.

Moreover, high EF24 levels have been associated with suppression of invasion and migration of HCC cells (by R. Zhao et al.), while high expression levels of Cadherin 17 have been associated with high frequency of bone marrow metastasis in a murine breast cancer model (by T. Okada et al.) and high expression of meiotic recombination 11 homolog A (MRE11) oncoprotein with clinical breast cancer progression (by C.-H. Yang et al.). The overall characterization of the primary tumor has been described for renal cell carcinoma by S. H. Kim et al., glioblastoma by W. Szopa et al., and nasopharyngeal carcinoma by A. Lu et al. The latter study includes the primary tumor immune-evaluation showing that in patients with elevated neutrophil to lymphocyte ratio (NLR) the balance is tipped in favor of tumor-promoting inflammation resulting in tumor cell proliferation and cancer metastasis.

Finally specific gene signatures have been proposed to optimize personalized cancer treatment; in particular the overall higher responsiveness to PARPi of patients carrying BRCA2 mutations has been reviewed by S. Murata et al. and specifically for pancreatic cancer by J. Martinez-Useros and J. Garcia-Foncillas.

We hope that our current paper collection will enrich our readers and researchers, giving them an overview of the current broad range of cancer biomarkers being pursued by research teams around the world.

*Franco M. Buonaguro* *Franco M. Buonaguro* *C. David Pauza* *C. David Pauza* *Maria Lina Tornesello* *Maria Lina Tornesello* *Pierre Hainaut* *Pierre Hainaut* *Renato Franco* *Renato Franco* *Massimo Tommasino* *Massimo Tommasino*

